# Medical Imaging Contrast Media Use

**DOI:** 10.1001/jamanetworkopen.2025.47304

**Published:** 2025-12-05

**Authors:** Florence X. Doo, Regent Lee, Andrea Rockall, Elizabeth Y. Rula, Linda Moy

**Affiliations:** 1 University of Maryland Medical Intelligent Imaging Center, Department of Diagnostic Radiology and Nuclear Medicine, University of Maryland School of Medicine, Baltimore; 2 University of Maryland Institute for Health Computing, North Bethesda; 3 Nuffield Department of Surgical Sciences, University of Oxford, Oxford, United Kingdom; 4 Department of Surgery and Cancer, Faculty of Medicine, Imperial College London, United Kingdom; 5 Department of Radiology, Imperial College Healthcare NHS Trust, London, United Kingdom; 6 Harvey L. Neiman Health Policy Institute, Reston, Virginia; 7 Department of Radiology, New York University Langone Medical Center, New York City

## Abstract

This cross-sectional study uses Medicare Part B claims data to examine trends in medical image contrast media use from 2011 to 2014.

## Introduction

According to the United Nations, the world is facing a triple planetary health crisis driven by climate change, pollution, and biodiversity loss. Within the health care sector, medical imaging contrast agents are an underrecognized contributor to this crisis.^1^ Both iodinated agents (used in computed tomography [CT] and angiography [CTA])^2^ and gadolinium-based agents (used in magnetic resonance imaging [MRI] and angiography [MRA])^3^ are excreted and incompletely removed by conventional wastewater treatment, and can accumulate as pollutants in aquatic ecosystems and in drinking water.^1^ This study quantifies contrast use patterns to potentially guide targeted strategies for health care sustainability.

## Methods

This cross-sectional study analyzed the US Medicare Part B fee-for-service limited public claims data (2011 to 2024 Physician/Supplier Procedure Summary), which provide national-level aggregated counts of billed procedures and represent approximately two-thirds of the Medicare beneficiary population, serving as a proxy for imaging procedural trends. Current Procedural Terminology codes were used to identify all imaging procedure types that used contrast (CT, CTA, MRI, or MRA). Estimated contrast volumes were calculated using standard doses (100 mL for iodinated agents; 15 mL for gadolinium-based agents). Analysis and descriptive statistics were performed using Python version 3.12.11 (Python Software Foundation). This was not human participants research as defined by 45 CFR 46, and thus, institutional review board approval was not pursued. We followed the STROBE reporting guideline.

## Results

From 2011 to 2024, Medicare beneficiaries underwent an estimated 169.3 million contrast-enhanced examinations across 82 CPT codes (41 iodinated [50%]: 28 CT [68%], 13 CTA [32%]; 41 gadolinium [50%]: 29 MRI [71%], 12 MRA [29%]), requiring approximately 13.5 billion mL of contrast media (Table). Iodinated agents dominated total combined contrast volume (12.9 billion [95.6%]), with CT abdomen or pelvis representing the largest single contributor at 4.4 billion mL. Among gadolinium agents, brain MRI led usage at 221 million mL. Annual contrast volume increased overall; following initial fluctuation (2011 to 2013), mean year-over-year growth from 2014 to 2019 was 5.2% for iodinated agents and 3.5% for gadolinium agents, with COVID-19 pandemic–related declines in 2020 (−9.6% for iodinated agents and −15.6% for gadolinium agents) followed by rebounds in 2021 (10.8% and 10.1%, respectively) before normalizing through 2024 (Figure A and B). By procedure count, the top 6 iodinated and 7 gadolinium examination types accounted for 80% or more of all contrast-enhanced procedures, demonstrating concentrated usage patterns (Figure C and D).

**Table.** Distribution of Contrast Media Usage by Procedure Type Among Medicare Part B Beneficiaries, 2011-2024^a^

CPT code	Procedure name	No. of procedures performed	Estimated contrast volume, mL
Iodinated (100 mL/ procedure)			
74177	CT abdomen and pelvis with contrast	44 223 409	4 422 340 900
71260	CT chest with contrast	26 532 050	2 653 205 000
71275	CTA chest (noncoronary) with contrast	16 804 380	1 680 438 000
74178	CT abdomen and pelvis without, then with contrast	8 481 132	848 113 200
70498	CTA neck with contrast	6 357 131	635 713 100
70496	CTA head with contrast	5 995 449	599 544 900
70491	CT soft tissue neck with contrast	3 734 253	373 425 300
74174	CTA abdomen and pelvis with contrast	3 473 431	347 343 100
74160	CT abdomen with contrast	1 639 178	163 917 800
74170	CT abdomen without, then with contrast	1 596 848	159 684 800
75635	CTA abdominal aorta and bilateral iliofemoral runoff	1 405 839	140 583 900
75574	CTA heart/coronary with 3D and quantitative assessment	1 250 345	125 034 500
71270	CT chest without, then with contrast	1150 003	115 000 300
72132	CT lumbar spine with contrast	814 346	81 434 600
75571	CTA heart with quantitative evaluation	726 602	72 660 200
70480	CT orbit, sella, or posterior fossa with contrast	717 989	71 798 900
74175	CTA abdomen with contrast	648 334	64 833 400
73701	CT lower extremity with contrast	536 068	53 606 800
72193	CT pelvis with contrast	474 193	47 419 300
75572	CTA heart for congenital anomalies	368 909	36 890 900
70460	CT head/brain with contrast	367 680	36 768 000
72129	CT thoracic spine with contrast	319 269	31 926 900
70487	CT maxillofacial with contrast	314 183	31 418 300
70492	CT soft tissue neck without, then with contrast	306 077	30 607 700
72126	CT cervical spine with contrast	236 928	23 692 800
73201	CT upper extremity with contrast	192 635	19 263 500
73706	CTA lower extremity with contrast	163 722	16 372 200
72191	CTA pelvis with contrast	143 858	14 385 800
74261	CT colonography with contrast	143 400	14 340 000
70481	CT orbit, sella, or posterior fossa with contrast	84 887	8 488 700
72194	CT pelvis without, then with contrast	64 718	6 471 800
70482	CT orbit, sella, or posterior fossa without, then with contrast	40 243	4 024 300
73206	CTA upper extremity with contrast	31 567	3 156 700
72133	CT lumbar spine without, then with contrast	31 290	3 129 000
73702	CT lower extremity without, then with contrast	29 841	2 984 100
70488	CT maxillofacial without, then with contrast	23 606	2 360 600
72127	CT cervical spine without, then with contrast	8164	816 400
73202	CT upper extremity without, then with contrast	6870	687 000
74262	CT colonography without, then with contrast	6282	628 200
75573	CTA heart for congenital anomalies with quantitative evaluation	3628	362 800
72130	CT thoracic spine without, then with contrast	3067	306 700
Total		129 451 804	12 945 180 400
Gadolinium (15 mL/procedure)			
70553	MRI brain without, then with contrast	14 752 491	221 287 365
74183	MRI abdomen without, then with contrast	4 659 016	69 885 240
70544	MRA head without contrast	4 300 587	64 508 805
72158	MRI lumbar spine without, then with contrast	3 644 453	54 666 795
72197	MRI pelvis without, then with contrast	2 692 992	40 394 880
72156	MRI cervical spine without, then with contrast	1 607 326	24 109 890
72157	MRI thoracic spine without, then with contrast	1 270 663	19 059 945
70547	MRA neck without contrast	1 200 916	18 013 740
70549	MRA neck without, then with contrast	943 710	14 155 650
70543	MRI orbit, face, and/or neck without, then with contrast	807 721	12 115 815
73720	MRI lower extremity without, then with contrast	759 260	11 388 900
77049	MRI breast without, then with contrast, bilateral	701 661	10 524 915
73723	MRI lower extremity joint without, then with contrast	342 894	5 143 410
70548	MRA neck with contrast	300 846	4 512 690
70552	MRI brain with contrast	273 808	4 107 120
73222	MRI upper extremity joint with contrast	255 597	3 833 955
70546	MRA head without, then with contrast	231 499	3 472 485
74185	MRA abdomen with or without contrast	197 238	2 958 570
73223	MRI upper extremity joint without, then with contrast	164 076	2 461 140
73220	MRI upper extremity without, then with contrast	149 366	2 240 490
73725	MRA lower extremity with or without contrast	127 582	1 913 730
71555	MRA chest with or without contrast	114 736	1 721 040
71552	MRI chest without, then with contrast	64 105	961 575
73722	MRI lower extremity joint with contrast	60 748	911 220
72149	MRI lumbar spine with contrast	49 197	737 955
74182	MRI abdomen with contrast	41 251	618 765
75563	Cardiac MRI for function with contrast	38 754	581 310
72198	MRA pelvis with or without contrast	36 404	546 060
70545	MRA head with contrast	33 932	508 980
72142	MRI cervical spine with contrast	24 619	369 285
72196	MRI pelvis with contrast	20 970	314 550
72147	MRI thoracic spine with contrast	17 819	267 285
77047	MRI breast with contrast	13 192	197 880
70542	MRI orbit, face, and/or neck with contrast	3639	54 585
73719	MRI lower extremity with contrast	3094	46 410
77048	MRI breast without, then with contrast, unilateral	1911	28 665
73225	MRA upper extremity with or without contrast	733	10 995
75559	Cardiac MRI for morphology with contrast	363	5445
72159	MRA spinal canal and contents with or without contrast	283	4245
71551	MRI chest with contrast	149	2235
73219	MRI upper extremity with contrast	41	615
Total		39 909 642	598 644 630
Combined total (iodinated and gadolinium-based contrast agents)		169 361 446	13 543 825 030

Abbreviations: CPT code, Current Procedural Terminology code; CT, computed tomography; CTA, computed tomography angiography; MRA, magnetic resonance angiography; MRI, magnetic resonance imaging.

^a^ Summary table displaying cumulative contrast use totals across the study period. Volume estimates assume 100 mL per iodinated contrast procedure and 15 mL per gadolinium-based contrast procedure.

**Figure.**
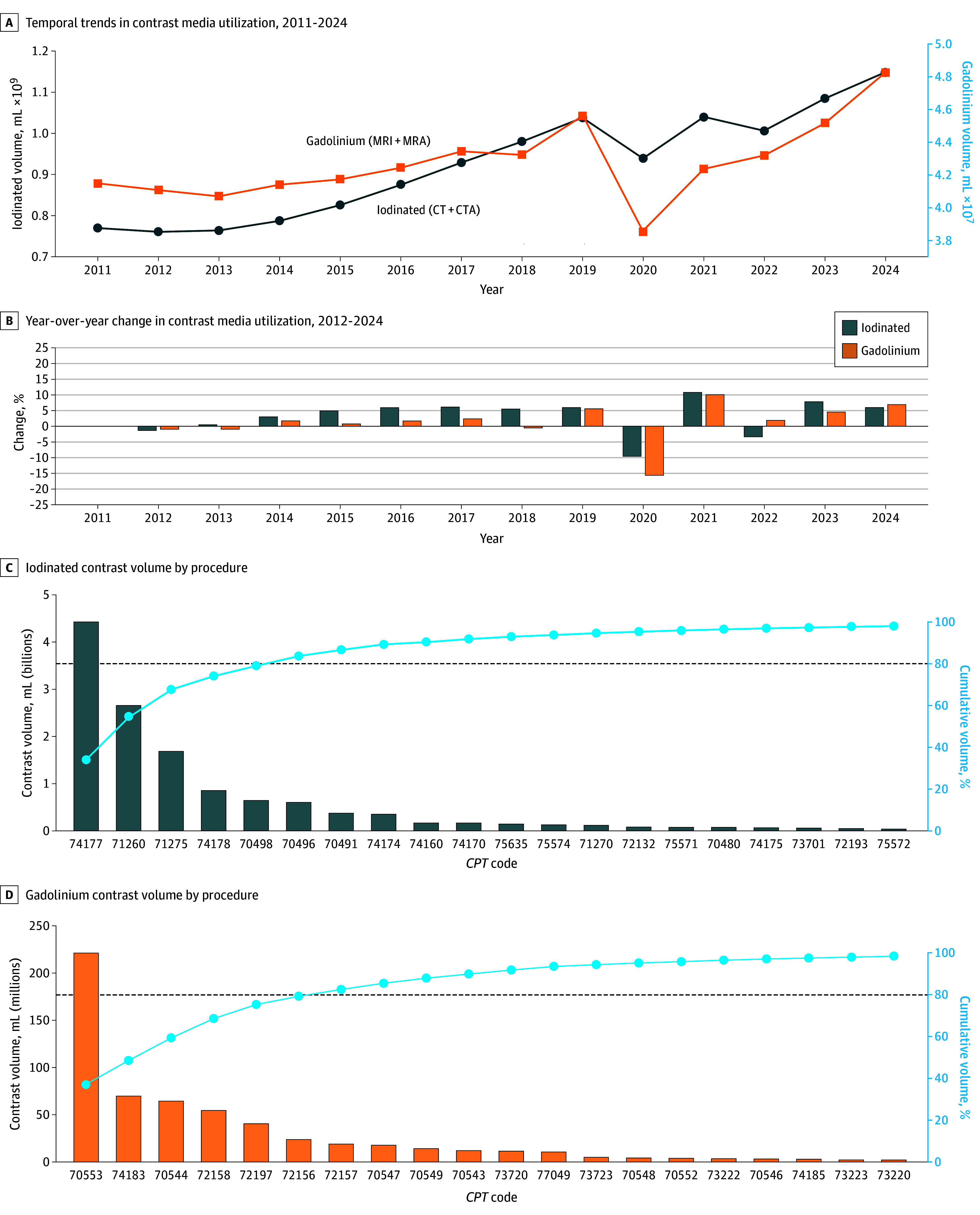
Trends in Contrast Media Usage Among US Medicare Part B Beneficiaries, 2011-2024 Panel A shows temporal trends in estimated annual iodinated (CT/CTA; left y-axis, black) and gadolinium-based (MRI/MRA; right y-axis, blue) contrast volumes, with year-over-year percent changes presented in Panel B. Pareto analysis of contrast volume distribution by CPT code for iodinated (C) and gadolinium-based (D) agents; bars represent estimated contrast volume (mL) by procedure, with cumulative contribution (blue line) identifying procedures that account for 80% or more of usage. CT accounted for 9.21 billion mL, CTA for 3.74 billion mL, MRI for 486.3 million mL, and MRA for 112.3 million mL, for a total of 13.5 billion mL of contrast material. *CPT, Current Procedural Terminology*; CT, computed tomography; CTA, computed tomography angiography; MRA, magnetic resonance angiography; MRI, magnetic resonance imaging.

## Discussion

This study quantifies the magnitude and trend of contrast media use in the US Medicare population, approximately 13.5 billion mL administered from 2011 to 2024, and shows that a subset of imaging procedures accounts for most of this volume. Because these agents have recognized environmental impacts,^1,2,3^ this concentrated use highlights actionable targets for sustainability efforts in health care.

Despite growing awareness, translation to clinical or policy change to mitigate contrasts’ planetary health risks remain limited.^4^ Both short- and long-term strategies must balance clinical quality, operational feasibility, and ecological responsibility. Incremental mitigation strategies include reducing inappropriate imaging orders, adopting weight-based dosing, and implementing recycling or multiuse vial systems.^5^ Emerging contrast alternatives like biodegradable CT or MRI agents and ultrasonography microbubbles, however, remain dependent on administered materials that continue generating injection-related waste from single-use needles, syringes, and tubing. Potentially transformative approaches like artificial intelligence-enabled virtual contrast imaging may eventually reduce contrast administration dependence.^6^ These methods are still experimental and require rigorous clinical evaluation and regulatory review, but if effective could simultaneously address chemical pollution, injection-related waste, and patient reaction risk and toxicity.

This study has limitations. Estimates are based on assumed standard contrast dosing and limited Medicare Part B aggregated data, which likely underestimate total US and global consumption. Nonetheless, these findings show the scale of contrast use and prompts the reimagination of contrast media in medical imaging. Contrast media are a measurable and substantial component of health care’s environmental footprint, and their stewardship should be integrated into broader strategies for climate-resilient, resource efficient, and high-quality patient care.

## Notes

Supplement.
Data Sharing Statement
