# Supplementary material for: Medical Imaging Contrast Media Use
Source: JAMA Netw Open. 2025 Dec 5;8(12):e2547304. doi: 10.1001/jamanetworkopen.2025.47304 (PMC12681031; doi:10.1001/jamanetworkopen.2025.47304)
Supplement: Supplement. — Data Sharing Statement [file jamanetwopen-e2547304-s001.pdf]

Data Sharing Statement

Doo. Medical Imaging Contrast Media Use. *JAMA Netw Open*. Published December 05, 2025.
doi:10.1001/jamanetworkopen.2025.47304

Data
Data available: No

Additional Information
Explanation for why data not available: Medicare Part B data already publicly available
